# PRMT1 expression in renal cell tumors- application in differential diagnosis and prognostic relevance

**DOI:** 10.1186/s13000-019-0901-6

**Published:** 2019-10-26

**Authors:** Jelena Filipović, Martina Bosić, Sanja Ćirović, Maja Životić, Duško Dunđerović, Dejan Đorđević, Snežana Živković-Perišić, Aleksandar Lipkovski, Jasmina Marković-Lipkovski

**Affiliations:** 1 Faculty of Medicine, Institute of Pathology, University of Belgrade, Dr. Subotića 1, Belgrade, Serbia; 2 Clinic for Urology, Clinical Center of Serbia, Faculty of Medicine, University of Belgrade, Belgrade, Serbia; 3 Institute of Public Health “Dr. Milan Jovanović Batut”, Belgrade, Serbia; 4 Faculty of Mathematics, University of Belgrade, Belgrade, Serbia

**Keywords:** PRMT1, EMT, Renal cell tumors, TCGA, GTEx

## Abstract

**Background:**

Protein arginine methyltransferase-1 (PRMT1) is associated with the progression of various tumor types and the process of epithelial to mesenchymal transition (EMT). However, the expression of PRMT1 in renal cell tumors (RCT) is unknown.

**Methods:**

We evaluated PRMT1 immunohistochemical (IHC) expression on tissue microarray (TMA) of 208 specimens of RCT, including clear cell renal cell carcinomas (ccRCC), papillary RCC type I and II (pRCC I and II), chromophobe RCC (chRCC), renal oncocytomas (RO), collecting duct carcinomas - Bellini (CDC) and multilocular cystic renal cell neoplasms of low malignant potential (MLCRN-LMP). Moreover, a subset of ccRCC, pRCC, chRCC, RO were also studied using conventional sections. PRMT1 expression in tumor tissue was compared to the IHC expression of EMT-related transcription factors (ZEB1, RUNX1, and TWIST1) and cell surface markers (ß-catenin, N- and E-cadherin). Additionally, qRT-PCR expression of PRMT1 in ccRCC, pRCC, and chRCC was evaluated and the results were compared to the mRNA PRMT1 transcript profiling data in The Cancer Genome Atlas (TCGA) and Genotype-Tissue Expression (GTEx) cohort.

**Results:**

PRMT1 immunoreactivity was observed in the majority of ccRCC, RO, all MLCRN-LMP, but in a minority of chRCC (*p* = 0.044), and it was associated with low grade and low stage ccRCC (*p* = 0.014; p = 0.044, respectively). ZEB1 immunoreactivity was noted in all RO, in minority of chRCC and neither of MLCRN-LMP (*p* < 0.001). The majority of PRMT1-negative ccRCC was negative to ZEB1 and showed cytoplasmic expression of TWIST1 (*p* = 0.028; *p* < 0.001, respectively). PRMT1 positive ccRCC mostly expressed RUNX1 (*p* = 0.019). PRMT1 and ZEB1 expression were associated with better cancer-specific survival in patients with ccRCC (*p* = 0.029; *p* = 0.009, respectively). In multivariate analysis, ZEB1 expression was an independent prognostic factor for cancer-specific survival (hazard ratio [HR], 0.367; *p* = 0.026). Significant IHC heterogeneity was observed in PRMT1, ZEB1 and TWIST1 expression (*p* < 0.001). Homogenous loss of PRMT1 was associated with high grade and high stage ccRCC, while the homogenous loss of PRMT1 and ZEB1 was more frequent in patients who died of ccRCC (*p* = 0.017; *p* = 0.040; *p* = 0.044; *p* = 0.009, respectively). Relative mRNA-PRMT1 expression in both cohorts was down-regulated in tumor tissue compared to non-tumor parenchyma (*p* = 0.009). Unlike in our samples, mRNA-PRMT1 expression in the TCGA cohort was not correlated to ccRCC tumor stage or grade. PRMT1, ZEB1, and TWIST1 expression were not associated with EMT related aberrant ß-catenin expression, a gain of N-cadherin or loss of E-cadherin expression. Only RUNX1 was associated with a gain of N-cadherin (*p* = 0.003).

**Conclusions:**

IHC expression of PRMT1 may be characteristic for low grade and low stage ccRCC, while the homogenous loss of PRMT1 may be significant for high grade and high stage ccRCC. Both, PRMT1 and/or ZEB1 expression, could be associated with better survival of the patients with ccRCC.

## Background

Renal cell tumors (RCT) comprise 80% of primary kidney tumors. Among RCT malignant types, clear cell RCC (ccRCC) accounts for 65–70%, papillary RCC (pRCC) 18.5%, chromophobe RCC (chRCC) 5–7%, and collecting duct carcinomas-Bellini (CDC) 1–2%. Renal oncocytoma (RO), as a benign tumor, comprises 5–9%, while MLCRN-LMP, as a borderline lesion, presents in less than 1% [1].

Deregulated gene expression, especially that related to epigenetic control mechanisms clearly contributes to the development of renal tumors [2, 3]. Epigenetic gene control includes methylation of DNA and/or histone proteins and regulation of miRNA signaling, without a change in DNA sequence [4]. Protein arginine methylation is crucial in the epigenetic regulation of gene transcription, mRNA splicing, DNA repair, protein cellular localization, and signaling processes [4]. Protein arginine methyltransferase 1 (PRMT1) is the predominant type I arginine methyltransferase in mammals, normally expressed in various tissues [4, 5]. Significant changes in expression of PRMT1 have been observed in various cancer types, such as lung carcinoma, colon cancer, breast carcinoma, prostate and bladder cancer, gastric carcinomas, and gliomas [6–14]. An interesting finding documented in previous studies is that PRMT1 can induce epithelial to mesenchymal transition (EMT), cellular senescence and apoptosis in various cancer cell types [15, 16]. EMT represents a switch from an epithelial phenotype to a mesenchymal phenotype, which results in increased cellular motility, decreased cellular interactions, and non-polarized cell organization. EMT is partially regulated by Wnt/β-catenin signaling pathway, whose hallmark is membrane dissociation of ß-catenin and its translocation into the cytoplasm or nucleus, which then leads to N-cadherin overexpression and reduced E-cadherin expression [17]. The function of PRMT1 in modulating EMT was suggested through the regulation of Zinc Finger E-Box Binding Homeobox 1 (ZEB1) in breast carcinoma and Twist Family BHLH Transcription Factor 1 (TWIST1) in non-small lung carcinoma (10). Furthermore, it was shown that PRMT1 directly methylates Runt-related transcription factor 1 (RUNX1) and functions as a co-activator for RUNX1-dependent transcriptional activation in different hematopoietic cell lineages [18].

The present study was designed to investigate PRMT1 expression in the renal parenchyma, to determine the diagnostic and prognostic significance of PRMT1 expression in various RCT types and to correlate its expression with EMT-related transcription factors: ZEB1 and TWIST1, EMT regulator RUNX1 and Wnt/β-catenin EMT signaling pathway-associated markers: ß-catenin, E-cadherin and N-cadherin.

## Methods

### Patient characteristics and tumor samples

A total of 208 patients with different types of RCT who underwent radical or partial nephrectomy between January 1st, 2010 and December 31st, 2016 at Clinic for Urology, Clinical Center of Serbia, were included in this retrospective study. Medical archival records were retrieved to obtain clinicopathological parameters including age, gender and tumor size (maximum tumor diameter). Descriptive characteristics of all 208 patients and histological characteristics are listed in Table 1. Most of the study group was composed of elderly male patients. Among all 208 patients, 178 (85.6%) underwent radical nephrectomy and 30 (14.4%) underwent partial nephrectomy. Tumor size ranged from 10 mm to 34 cm in the largest diameter, both in patients with ccRCC. Sarcomatoid change was observed in 2 ccRCC with nuclear grade IV. Information about patients’ outcomes, including the time between nephrectomy and cancer-related death or last follow-up (if death did not occur), was also recorded. The median follow-up time was 44.2 months, ranging from 1 month in a patient with chRCC to 94 months in a patient with RO. For 14 (6.7%) patients with RO, survival data wasn’t available.

**Table 1:** Patients’ demographics and tumor characteristics

	Histopathological types of RCT							
	ccRCC	pRCC I	pRCC II	chRCC	RO	CDC	MLCRN-LMP	Total N (%)
**N** (%)	120 (57.7)	7 (3.4)	16 (7.7)	28 (13.5)	25 (11.0)	7 (3.4)	5 (2.4)	208 (100)
Age, mean ± SD (years)	61.6 ± 9.05	70.4 ± 11.01	62.6 ± 10.8	58.3 ± 13.2	58.5 ± 12.5	52.7 ± 10.7	51.8 ± 15.9	60.7 ± 10.7
Gender N (%)								
Male	79 (65.8)	6 (85.7)	10 (62.5)	15 (53.6)	12 (48.0)	6 (85.7)	4 (80.0)	132 (63.5) (64.4)
Female	41 (34.2)	1 (14.3)	6 (37.5)	13 (46.4)	10 (40.0)	1 (14.3)	1 (20.0)	73 (35.1)
Tumor size, mean ± SD (mm)	69.9 ± 43.8	78.3 ± 46.3	74.8 ± 22.7	65.4 ± 28.8	54.2 ± 29.5	66.4 ± 29.7	49.2 ± 12.1	68 ± 38.2
Grade N (%)								
I	21 (17.5)	2 (28.6)	0 (0)	N/A	N/A	N/A	N/A	31 (18.2)
II	68 (56.7)	5 (71.4)	8 (50.0)					97 (56.7)
III	18 (15.0)	0 (0)	4 (25.0)					25 (14.6)
IV	13 (10.8)	0 (0)	4 (25.0)					18 (10.5)
Stage N (%)								
pT1	58 (48.3)	4 (57.1)	5 (31.3)	13 (46.4)	N/A	N/A	N/A	80 (39.4)
pT2	13 (10.8)	1 (14.3)	3 (18.8)	3 (10.7)				20 (9.6)
pT3	38 (31.7)	2 (28.6)	7 (43.8)	10 (35.7)				57 (29.3)
pT4	11 (9.2)	0 (0)	1 (6.3)	2 (7.1)				14 (7.7)
PN	15 (12.5)	2 (28.6)	3 (18.6)	4 (14.3)	4 (36.4)	0 (0)	2 (40.0)	30 (14.4)
Follow up, mean months	41.7	50.3	41.9	49.3	54.4	36.1	74.8	44.2
Disease related death N (%)	39 (81.2)	0 (0.0)	5 (10.4)	2 (4.1)	0 (0)	2 (4.1)	0 (0)	48 (98.0)

Abbreviations: RCT, Renal cell tumors; ccRCC,clear cell renal cell carcinomas; pRCC I,papillary renal cell carcinoma type I; pRCC II, papillary renal cell carcinoma type II; chRCC, chromophobe renal cell carcinoma; RO, renal oncocytoma; CDC,collecting duct carcinoma-Bellini; MLCRN-LMP, multilocular cystic renal neoplasm of low malignant potential; N, number; SD, standard deviation; N/A, not applicable; ISUP (International Society of Urological Pathology) nuclear grading system and updated American Joint Committee on Cancer (AJCC) tumor–node–metastasis (TNM) classification 8th edition for tumor staging were used

### Pathological evaluation criteria

The diagnosis of RCT types was confirmed during the final pathological assessment, using the 4th edition of WHO Classification of Tumors of the Urinary System and Male Genital Organs [1]. These types of RCT included 120 (57.7%) ccRCC, 7 (3.4%) pRCC I, 16 (7.7%) pRCC II, 28 (13.5%) chRCC, 25 (12.0%) RO, 7 (3.4%) CDC, and 5 (2.4%) MLCRN-LMP. Each case was evaluated regarding the nuclear grade, pathologic stage of the disease, and tumor size. Nuclear grading for ccRCC and pRCC was revised based on the description by WHO/ISUP, 2016 [1]. Since there are not clear recommendations for grading chRCC and CDCs, they were not graded [19]. Tumor staging was evaluated according to the updated American Joint Committee on Cancer (AJCC) tumor–node–metastasis (TNM) classification 8th edition, by reviewing the gross description and representative slides [20].

### TMA

TMA was constructed manually. As TMA only contains a limited amount of tissue, we were aware of the possible variation of immunostaining due to tissue heterogeneity. To provide the high accuracy of our results in the representation of the whole section, we sampled three different cores 1.2 mm from each specimen, as it was is recommended previously [21]. The hematoxylin-eosin (H&E) stained slides were examined and the three different the most high-grade areas (subcapsular, middle and periphery of the tumor), were marked by pathologists. Microarray samples were punched out from the selected regions of each donor block and transferred into a new recipient paraffin block using a hollow needle with an inner diameter of 1,2 mm. An additional core of normal-appearing kidney cortex and medulla was taken randomly for each case. Five-micrometer sections were cut from completed TMA blocks and transferred to adhesive slides to be used for immunohistochemical staining. Thus, 5 μm thickness sections of each tissue array block were stained by H&E to confirm the grade and histological type of each tissue core spot.

### Conventional sections

In order to validate our methodology and to increase the confidence in our results, we used the corresponding whole-mount sections in a subset of 45 cases used for TMA. The selected cohort included: 15 ccRCC, 3 pRCC I, 7 pRCC II, 10 chRCC, and 10 RO. ccRCC and pRCC were chosen according to different nuclear grades. Seven cases of ccRCC were in low grade, eight cases were in high grade, including one with sarcomatoid features. In addition, we selected five cases from each of low- and high-grade pRCC. ChRCC and RO were taken randomly. Five-micrometer sections were cut from Formalin-Fixed Paraffin-Embedded (FFPE) tissue blocks of each case and transferred to adhesive slides for immunohistochemical staining.

### Immunohistochemistry

All TMA and conventional sections were deparaffinized at 60 °C for 20 min and rehydrated in graded ethanols. Antigen retrieval was performed by immersing the tissues in citrate buffer (pH = 6.0) for 20 min in a water bath. Endogenous peroxidase and non-specific staining were blocked with 3% H_2_O_2_ for 20 min at room temperature. The TMA sections were incubated for 1 hour at room temperature with the following primary antibodies: PRMT1 (ab92299, Abcam, dilution: 1/200), ZEB1 (HPA027524, Sigma Aldrich, dilution: 1/500), RUNX1 (sc-365,644, Santa Cruz, dilution: 1/50), TWIST1 (ABD29 Merck Millipore, dilution: 1/100), E-cadherin (spm471, Santa Cruz, dilution: 1/100), N-cadherin (6D11, DAKO, dilution: 1/100), ß-catenin (ß-catenin-1, Dako, dilution: 1/100). Whole-mount sections were incubated with the PRMT1 (ab92299, Abcam, dilution: 1/200) and ZEB1 (HPA027524, Sigma Aldrich, dilution: 1/500). All slides were then incubated with Envision ^in^ FLEX/HRP (K4063, Dako, Denmark) for 30 min. Staining patterns were visualized by exposure to 3,3′-diaminobenzidine (DAB) and counterstained with Mayer’s hematoxylin. Finally, the slides were dehydrated in ethanol, cleared in xylene and mounted for examination. Human kidney tissue was used as a positive control of staining and replacement of the primary antibody with Tris-buffered saline was used as a negative control.

### Evaluation of immunostaining

Immunostaining of all markers was independently evaluated by three pathologists (J.F. M.B. M.Ž.), who were blinded to the patient outcome and pathological information. Valid immunoreactivity was considered as following: nuclear staining of PRMT1, ZEB1 and RUNX1, cytoplasmic and/or nuclear for TWIST1, membranous for N- and E-cadherin, while for ß-catenin, membranous staining was considered to be normal and nuclear and/or cytoplasmic immunopositivity was aberrant. Tumors were considered positive when more than 5% of tumor cells expressed positive staining to all analyzed markers, with at least moderate intensity, and at least in one core. In addition, the analysis of IHC heterogeneity was performed. If the staining pattern was equal among the cores, it was grouped as a homogenous negative or homogenous positive. The group with different immunopositivity among the cores was considered as heterogenous. In addition, PRMT1 expression in normal renal parenchyma was examined. Moreover, PRMT1 and ZEB1 expression in a subset of TMA cases were compared to corresponding whole-mount sections. We analyzed whether homogenous negative, homogenous positive or heterogeneous staining on TMA corresponds to the same staining pattern in subcapsular, middle or peripheral areas on whole-mount sections. A consensus was achieved for all samples. The slides were evaluated using the light microscope BX53 with DP12-CCD camera (Olympus, Germany).

### RNA isolation and qRT-PCR analysis of mRNA PRMT1

Tissues were collected on surgery and kept in liquid nitrogen. Fresh samples of 5 ccRCC, 2 pRCC and 1 chRCC with adjacent normal renal tissue were analyzed in triplicates. Tissue lysis was obtained in lysis buffer in a Tissue Lyser LT Adapter (Qiagen, United Kingdom) for 5 minutes. A TRIzol Reagent (Takara, Shiga, Japan) and Minipipet angio Kit (Isto Takara) were used for total RNA extraction in accordance with the manufacturer’s protocol. The quantity of the isolated RNA was assessed using NanoDrop 2000 spectrophotometer (Thermo Scientific, Germany). 100 ng of total RNA was digested with DNaseI (Sigma Aldrich, United Kingdom) and used for cDNA synthesis using Super Script II Reverse Transcriptase (Life Technologies, Germany). For quantitative real-time reverse transcription PCR (qRT-PCR) analysis, diluted cDNA (1/10) was used as a template in a Fast SYBR Green Master Mix (Life Technologies, Germany) and run on Step One Plus Real-Time PCR System (Applied Biosystems) in a total reaction volume of 20 μl. Primers for PRMT1 gene (forward, 5′-ATCTCGCACCACACCTTCTA-3′; reverse, 5′-CGGTATAGATGTCCACCTCCTTTATG − 3′), were designed and purchased from Primer Design.

### TCGA and GTEx cohort

mRNA PRMT1 transcript profiling data in Fragment Per Kilo Base Million (FPKM) values of 877 tumor samples in The Cancer Genome Atlas (TCGA) cohorts and 32 samples of normal tissue in Genotype-Tissue Expression (GTEx) cohort were downloaded from the Human Protein Atlas data portal (https://www.proteinatlas.org/) in April 2019. Patients clinicopathological data are collected from the TCGA resource network, as in [22]. Merged data are available in Additional file 2: Table S1. Patient characteristics are summarized in Additional file 3: Table S2. Overall, 60,1% of patients (*n* = 528) were diagnosed as ccRCC; 23,5% (*n* = 285) were classified as pRCC and 7,3% (*n* = 64), were classified as chRCC. Approximately 61% of the patients were male. mRNA PRMT1 expression level was analyzed in cancerous and normal renal parenchyma. Furthermore, mRNA PRMT1 expression level in ccRCC, pRCC and chRCC was compared to tumor stage, while tumor grade was available only for ccRCC.

### Statistical analysis

The results are presented as count (percent) or mean ± standard deviation. T-test, ANOVA, Mann-Whitney U test, Pearson chi-square test, Fisher’s exact test, and Kruskal Wallis test were used as appropriate. Kaplan Meier and Cox regression were used for survival analysis. All *p* values less than 0.05 were considered significant. All data were analyzed using SPSS 20.0 (IBM corp.) statistical software.

## Results

### Expression of PRMT 1 in normal kidney parenchyma

**Fig. 1:**
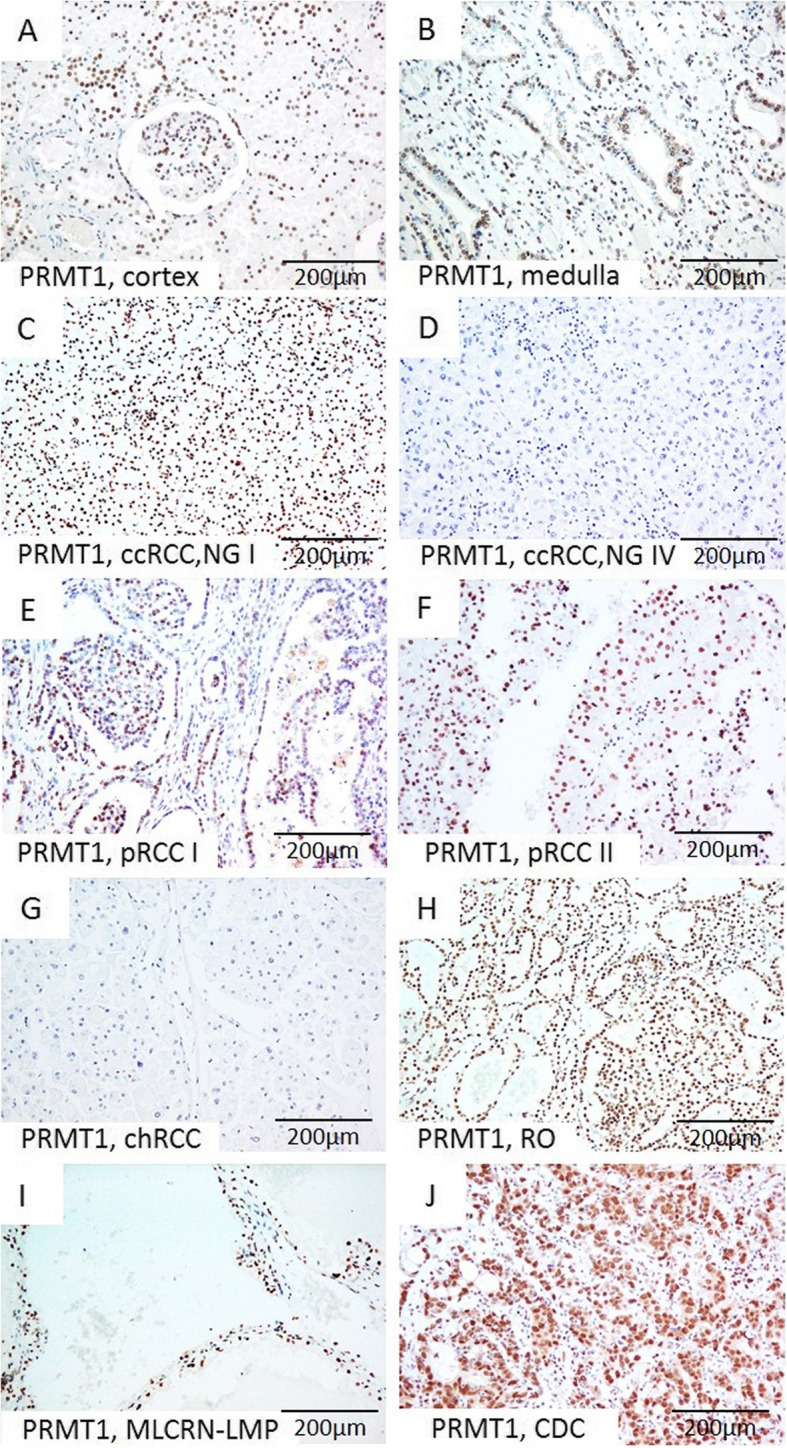
Representative microscopic photographs of PRMT1 expression in non-tumor renal parenchyma (**a**-**b**) and various RCT (**c**-**j**). **(a**) Cortex, (**b**) Medulla; (**c**) Diffuse strong nuclear positivity in low-grade ccRCC, (**d**) Negative immunostaining in high grade ccRCC; (**e**-**f**) Strong nuclear expression in pRCC, type I and pRCC, type II, respectively, (**g**) Absence of expression in chRCC, in contrast to (**h**) strong diffuse nuclear positivity in RO, (**i**-**j**) Positive staining in MLCRN-LMP and CDC, respectively. Original magnification, ×200. Abbreviation: NG-nuclear grade

We observed nuclear PRMT1 IHC expression in all samples of the renal parenchyma used as a positive control. PRMT1 was expressed in various cortical structures, such as epithelial cells of proximal and distal tubules, glomerular mesangial cells and parietal cells of Bowman’s capsule. In the medulla, PRMT1 was observed in epithelial cells of collecting ducts (Fig. 1 a-b).

### PRMT 1, ZEB 1, RUNX 1, and TWIST 1 expression in RCT types

**Fig. 2:**
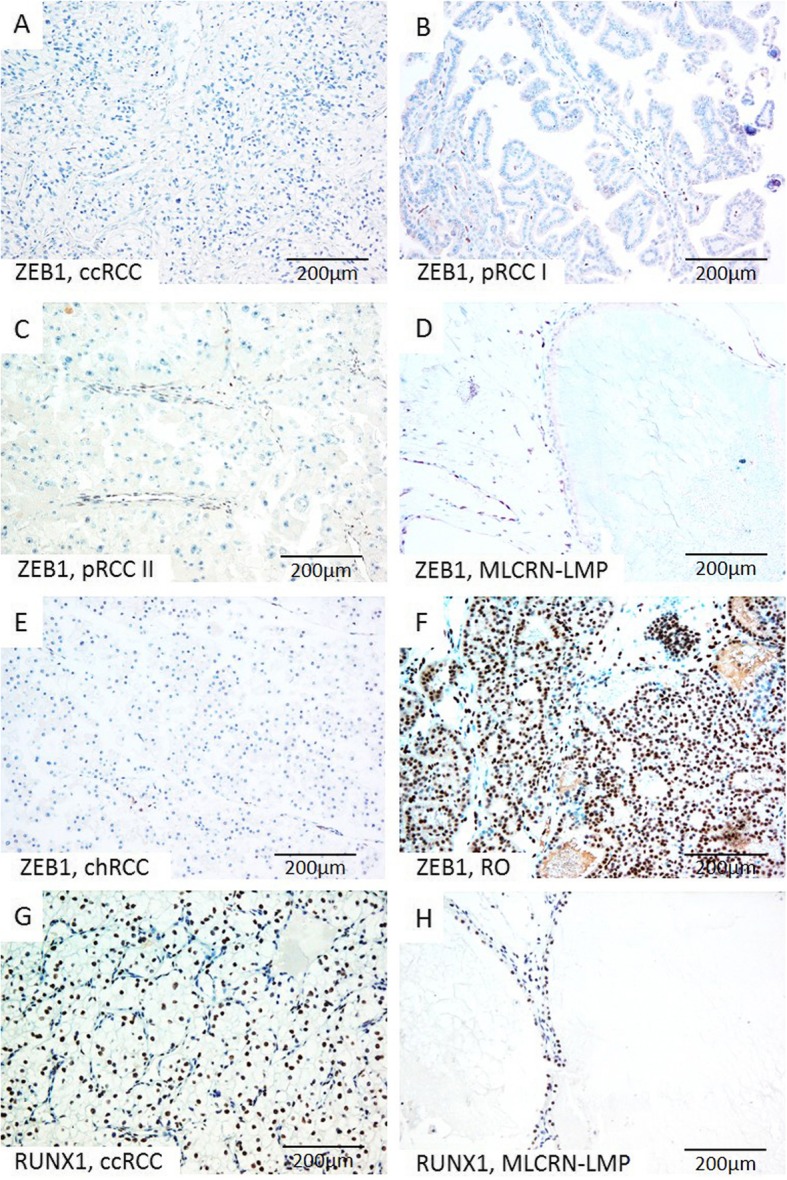
Representative microscopic photographs of ZEB1 (**a**-**f**) and RUNX1 (**g**-**h**) expression in various RCT. (**a**-**f**) Absence of ZEB1 on tumor cells, but positive nuclear staining on tumor blood vessels in ccRCC, pRCC type I, pRCC type II, MLCRN-LMP and chRCC, (**f**) in contrast to strong diffuse nuclear expression of ZEB1 on tumor cells in RO; (**g**-**h**) Strong and diffuse nuclear RUNX1 expression on tumor cells immunopositivity in ccRCC and MLCRN-LMP, respectively. Original magnification, × 200

**Fig. 3:**
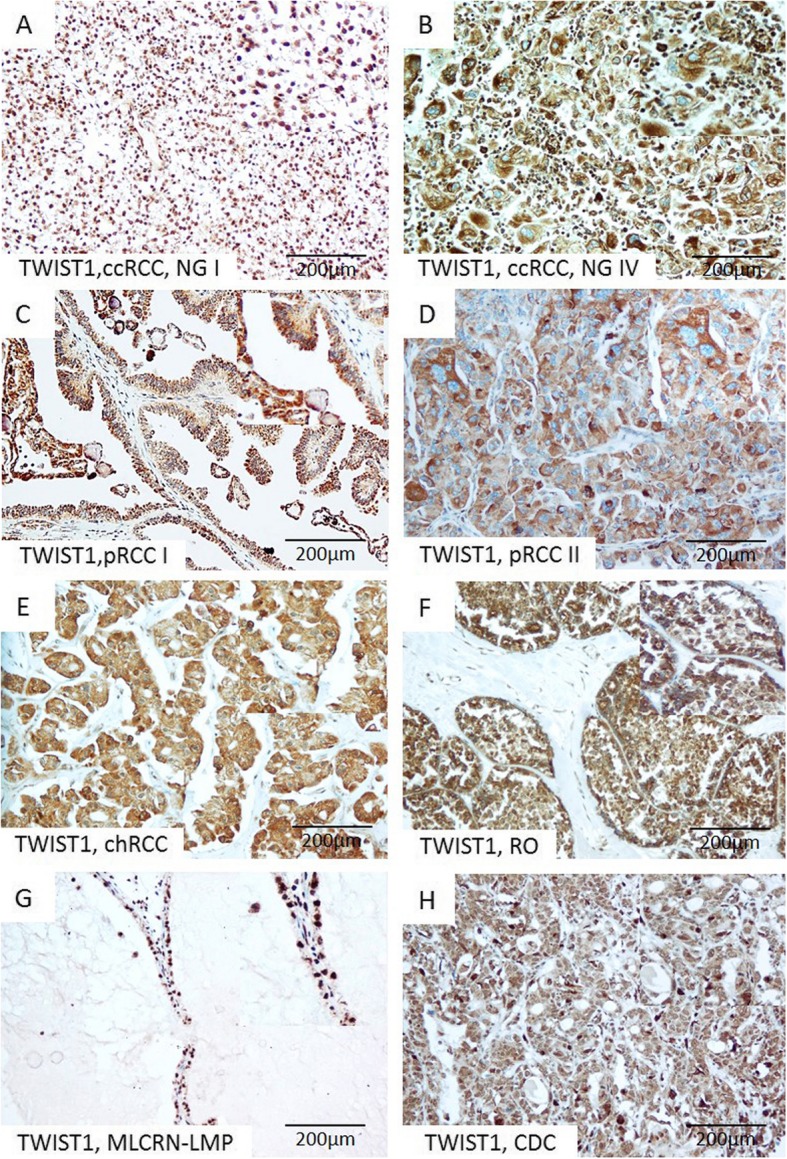
Representative microscopic photographs of TWIST1 expression in various RCT. (**a**) Strong diffuse nuclear expression in low-grade ccRCC, (**b**) Cytoplasmic immunopositivity in high-grade ccRCC, (**c**–**f**) Cytoplasmic immunopositivity in pRCC, type I, pRCC, type II, chRCC and RO, respectively, (**g**–**h**) Nuclear expression in MLCRN-LMP and CDC, respectively. Original magnification, × 200. Abbreviation: NG-nuclear grade

Immunohistochemical expression of PRMT1, ZEB1, RUNX1, and TWIST1 in different tumor types is summarized in Table 2. Expression patterns of PRMT1 and ZEB1 immunopositivity were associated with different RCT types (*p* values 0.044 and < 0.001, respectively) (Table 2). PRMT1 expression was observed in all analyzed tumor types, notably in the majority of ccRCC and pRCC, almost all RO, all MLCRN-LMP, and in minority of chRCC (Fig. 1 c-j). ZEB1 was negative in a majority of ccRCC and pRCC and in all MLCRN-LMP (Fig. 2 a-d.) ZEB1 was negative in a majority of chRCC, but positive in almost all RO cases (Fig. 2 e-f.). RUNX1 immunopositivity was noted in all tumor types, notably in a majority of ccRCC and in all MLCRN-LMP, however, without statistical significance (Fig. 2 g-h). TWIST1 was also observed in all tumor types, mostly with cytoplasmic immunopositivity and occasionally with nuclear expression, but without statistical significance (Fig. 3 a-h). Statistically significant differences in co-expression of PRMT1 with ZEB1, RUNX1 and TWIST1 were observed only in ccRCC, but not in other analyzed tumor types (Table 3). The majority of PRMT1 negative ccRCC showed mutual loss of ZEB1 (*p* = 0.028). On the other hand, the majority of PRMT1 positive cases expressed RUNX1 (*p* = 0.019). Also, the majority of PRMT1 negative ccRCC showed solely cytoplasmic expression of TWIST1 without nuclear positivity (*p* < 0.001).

**Table 2:** Immunohistochemical positivity of PRMT1, ZEB1, RUNX1 and TWIST1 among different tumor types

Histological subtype	PRMT1		ZEB1		RUNX1		TWIST1	
	+	–	+	–	+	–	+	–
ccRCC	73/120 (60.8)	47/120 (39.2)	37/113 (32.7)	76/113 (67.3)	70/117 (59.8)	47/117 (40.2)	112/115 (97.4)	3/115 (2.6)
pRCC I	4/7 (57.1)	3/7 (42.9)	0/7 (0)	7/7 (100)	2/7 (28.6)	5/7 (71.4)	7/7 (100)	0/7 (0.0)
pRCC II	12/16 (75.0)	4/16 (25.0)	1/16 (6.6)	14/15 (93.4)	7/16 (43.8)	9/16 (56.3)	16/16 (100)	0/16 (0.0)
chRCC	12/28 (42.8)	16/28 (57.2)	2/27 (7.1)	25/27 (92.9)	13/28 (46.4)	15/28 (53.6)	28/28 (100)	0/28 (0.0)
RO	24/25 (96.1)	1/25 (9.4)	24/25 (96.0)	1/25 (4.0)	9/25 (36.0)	16/25 (64.0)	25/25 (100)	0/25 (0.0)
CDC	5/7 (71.4)	2/7 (28.6)	3/7 (42.9)	4/7 (57.1)	3/7 (42.9)	4/7 (57.1)	7/7 (100)	0/7 (0.0)
MLCRN-LMP	5/5 (100)	0/0 (0.0)	0/5 (0)	5/5 (100)	5/5 (100)	0/5 (0.0)	5/5 (100)	0/5 (0.0)
*P* value	0.044*		<0.001*		0.149		0.897	

Abbreviations: PRMT1, protein arginine methyltransferase 1; ZEB1, Zinc Finger E-Box Binding Homeobox 1; RUNX1, Runt-related transcription factor 1; TWIST1, Twist Family BHLH Transcription Factor 1; ccRCC, clear cell renal cell carcinomas; pRCC I, papillary renal cell carcinoma type I; pRCC II, papillary renal cell carcinoma type I; chRCC, chromophobe renal cell carcinoma;CDC, collecting duct carcinomas-Bellini; MLCRN-LMP, multilocular cystic renal neoplasm of low malignant potential; +, positive; −, negative

*Statistically significant *p*-value

**Table 3:** Evaluation of co-expression patterns of PRMT1 and other transcription factors ZEB1, RUNX1, and TWIST1 in ccRCC

IHC marker (total cases)	PRMT1		*P* value
	positive	negative	
ZEB1 (113)			
positive	27 (40.9)	10 (21.3)	0.028*
negative	39 (59.1)	37 (78.7)	
RUNX1 (117)			
positive	48 (68.6)	22 (46.8)	0.019*
negative	22 (31.4)	25 (53.2)	
TWIST1 (115)			
nuclear ± cytoplasmic	37 (53.6)	10 (21.7)	<0.001*
cytoplasmic	32 (46.4)	36 (78.3)	

Abbreviations: PRMT1, protein arginine methyltransferase 1; ZEB1, Zinc Finger E-Box Binding Homeobox 1; RUNX1, Runt-related transcription factor 1; TWIST1, Twist Family BHLH Transcription Factor 1; ccRCC, clear cell renal cell carcinomas

*Statistically significant *p*-value

### The heterogeneity of PRMT1 and ZEB1 IHC expression in RCT types

The significant heterogeneity of PRMT1, ZEB1, and TWIST1 immunostaining was observed among analyzed tumor types (*p* < 0.001) (Table 4). Thus, PRMT1 expression among ccRCC was mostly heterogenous, while ZEB1 was mainly homogenous negative (Additional file 1: Figure S1A-F). PRMT1 and ZEB1 were more frequently homogenous negative in chRCC (Additional file 1: Figure S1G-L). RO the most frequently expressed PRMT1 homogenously, while ZEB1 positivity was mostly heterogenous (Additional file 1: Figure S1M-R). TWIST1 in the majority of cases was heterogenous or homogenous positive (Table 4).

**Table 4:** Heterogeneity of PRMT1, ZEB1, RUNX1 and TWIST1 immunohistochemical expression among different tumor types

Histological subtype	PRMT1			ZEB1			RUNX1				TWIST1	
	+	+/−	–	+	+/−	–	+	+/−	–	+	−/+	–
ccRCC	24 (20.0)	49 (40.8)	47 (39.2)	4 (3.5)	32 (27.8)	79 (68.7)	12 (10.2)	58 (49.6)	47 (40.2)	51 (43.6)	63 (53.8)	3 (2.6)
pRCC I	2 (28.6)	2 (28.6)	3 (42.9)	0 (0.0)	0 (0.0)	7 (100)	0 (0.0)	2 (28.6)	5 (71.4)	4 (57.2)	3 (42.8)	0 (0.0)
pRCC II	3 (18.8)	9 (56.2)	4 (25.0)	0 (0.0)	1 (6.3)	15 (93.8)	0 (0.0)	7 (43.7)	9 (56.3)	11 (64.6)	5 (29.4)	0 (0.0)
chRCC	7 (25.0)	5 (17.9)	16 (57.1)	0 (0.0)	2 (7.4)	25 (92.6)	0 (0.0)	13 (48.2)	14 (51.8)	20 (74.1)	7 (25.9)	0 (0.0)
RO	18 (72.0)	6 (24.0)	1 (4.0)	6 (24.0)	18 (72.0)	1 (4.0)	0 (0.0)	9 (36.0)	16 (64.0)	25 (100)	0 (0.0)	0 (0.0)
CDC	3 (42.9)	2 (28.6)	2 (28.6)	0 (0.0)	3 (42.9)	4 (57.1)	0 (0.0)	3 (60.0)	2 (40.0)	5 (83.3)	1 (16.7)	0 (0.0)
MLCRN-LMP	3 (60.0)	2 (40.0)	0 (0.0)	0 (0.0)	0 (0.0)	5 (100)	1 (20.0)	3 (60.0)	1 (20.0)	3 (60.0)	2 (40.0)	0 (0.0)
*P* value	*p* < 0.001*				*p* < 0.001*		*p* = 0.183			*p* < 0.001*		

Abbreviations: PRMT1, protein arginine methyltransferase 1; ZEB1, Zinc Finger E-Box Binding Homeobox 1; RUNX1, Runt-related transcription factor 1; TWIST1, Twist Family BHLH Transcription Factor 1; ccRCC, clear cell renal cell carcinomas; pRCC I, papillary renal cell carcinoma type I; pRCC II, papillary renal cell carcinoma type I; chRCC, chromophobe renal cell carcinoma;CDC, collecting duct carcinomas-Bellini; MLCRN-LMP, multilocular cystic renal neoplasm of low malignant potential; +, homogenous positive; +/− heterogenous;-, homogenous negative

*Statistically significant *p*-value

### PRMT1 and ZEB1 IHC expression on conventional sections

Homogenous positive or homogenous negative PRMT1 and ZEB1 expression on TMA, corresponded to the same staining pattern on whole-mount section, regardless of the tumor type or nuclear grade. Heterogenous PRMT1 and ZEB1 expression on TMA corresponded to the same subcapsular and occasional peripheral and/or middle areas on analyzed conventional sections. One case of ccRCC with sarcomatoid features showed mutual loss of PRMT1 and ZEB1 on TMA and whole-mount section (Additional file 4: Table S3).

### Association of PRMT1, RUNX1, ZEB1 and TWIST 1 with clinicopathological parameters in various types of RCT

As clinical behavior of tumor stage pT1 is similar to pT2, and pT3 to pT4, we analyzed the significance of PRMT1, RUNX1, ZEB1 and TWIST 1 expression in pT1/pT2 versus pT3/pT4 stage group. Likewise, tumor grades were grouped as low grade (I/II) and high grade (III/IV) [23]. Analysis of expression profiles showed association only of PRMT1 expression with clinic-pathological parameters in ccRCC patients (Table 5). PRMT1 expression was often present in low-grade ccRCC and low stage ccRCC (*p* = 0.014, *p* = 0.044, respectively). Moreover, homogenous loss of PRMT1 was observed in 58% high grade and in 49% of high stage ccRCCs (*p* = 0.017, *p* = 0.040, respectively) (Table 6). Interestingly, two cases of ccRCC with sarcomatoid change showed homogenous loss to PRMT1 and ZEB1, heterogenous positivity to RUNX1 and cytoplasmic TWIST1 expression. Gender and age of ccRCC patients were not associated with PRMT1 expression. Further, RUNX1, ZEB1 or TWIST1 expression didn’t show association with clinicopathological features in any of the analyzed tumor types.

**Table 5:** The comparative analysis of PRMT1, ZEB1, RUNX1 and TWIST1 immunopositivity with clinic-pathological features of the patients with ccRCC

	PRMT1		*p*-value	ZEB1		*p*-value	RUNX		*p*-value	TWIST1		*p*-value
	+	–		+	–		+	–		+	–	
Grade												
I/II	62 (68.8)	28 (31.2)	0.009*	38 (35.7)	54 (64.3)	0.093	51 (59.3)	35 (40.7)	0.846	84 (97.7)	2 (2.3)	1
III/IV	13 (41.9)	18 (58.1)		6 (19.4)	25 (80.6)		19 (61.3)	12 (38.7)		30 (96.8)	1 (3.2)	
Stage												
pT1/pT2	49 (69.0)	22 (31.0)	0.046*	23 (34.3)	44 (65.7)	0.409	37 (54.4)	31 (45.6)	0.159	67 (97.1)	2 (2.9)	
pT3/pT4	25 (51.0)	24 (49.0)		13 (27.1)	35 (72.9)		33 (67.3)	16 (32.7)		47 (97.9)	1 (2.9)	1
Gender												
Male	44 (55.7)	35 (44.3)	0.110	23 (30.7)	52 (69.3)	0.509	46 (60.5)	30 (39.5)	0.834	76 (98.8)	1 (1.2)	0.276
Female	29 (70.7)	12 (29.3)		14 (36.8)	24 (63.2)		24 (58.5)	17 (41.5)		39 (95.1)	2 (4.9)	
Age, mean ± SD (years)	61 ± 9.45	62.6 ± 8.38	0.346	63 ± 8.75	61.2 ± 9	0.378	62 ± 8.9	61 ± 8.7	0.704	61.9 ± 8.8	59 ± 5.03	0.536
Tumour size, mean ± SD (mm)	67 ± 48.75	74.5 ± 32.3	0.362	63 ± 35.1	74.1 ± 45.7	0.181	70 ± 50	71.7 ± 32.8	0.830	70.6 ± 43.7	65 ± 18.03	0.823

Abbreviations: PRMT1, protein arginine methyltransferase 1; ZEB1, Zinc Finger E-Box Binding Homeobox 1; RUNX1, Runt-related transcription factor 1; TWIST1, Twist Family BHLH Transcription Factor 1; ccRCC-clear cell renal cell carcinomas; SD, standard deviation;

ISUP (International Society of Urological Pathology) nuclear grading system and updated American Joint Committee on Cancer (AJCC) tumor–node–metastasis (TNM) classification 8th edition for tumor staging were used

*Statistically significant *p*-value

**Table 6:** Immunohistochemical heterogeneity of PRMT1 in ccRCC with different nuclear grade and stage

	PRMT1			*p*-value
	homogenous positive	heterogeneous	homogenous negative	
Grade				
I/II	22 (24.5)	40 (44.4)	28 (31.1)	0.017*
III/IV	2 (6.5)	11 (35.5)	18 (58.0)	
Stage				
pT1/pT2	19 (26.8)	30 (42.3)	22 (31.0)	0.040*
pT3/pT4	5 (10.2)	20 (40.8)	24 (49.0)	

Abbreviation: PRMT1, protein arginine methyltransferase 1

ISUP (International Society of Urological Pathology) nuclear grading system and updated American Joint Committee on Cancer (AJCC) tumor–node–metastasis (TNM) classification 8th edition for tumor staging were used. *Statistically significant *p*-value

### Association of PRMT1, RUNX1, ZEB1 and TWIST 1 with markers of EMT

Among analyzed RCT types, aberrant ß-catenin cytoplasmic immunoreactivity was observed in 147 (71.7%) of cases, without nuclear positivity. N-cadherin expression was observed in 123 (59.1%) cases, while the loss of E-cadherin immunopositivity was detected in 41 (19.7%) cases. PRMT1 expression was associated neither with aberrant ß-catenin expression nor with N- cadherin gain or E-cadherin loss in analyzed tumor types. However, the association between RUNX1, N-and E-cadherin expression was significant only in ccRCC (Table 7). The majority of RUNX1 positive ccRCC expressed N-cadherin (*p* = 0.003). E-cadherin loss was less frequent in RUNX1 positive than in RUNX1 negative ccRCC (*p* = 0.019).

**Table 7:** Association of PRMT1, ZEB1, RUNX1, and TWIST1 with WNT-β-catenin/EMT hallmarks (β-catenin, N- and E-cadherin) in ccRCC

Transcription factor	Wnt-β-catenin/EMT hallmarks					
	Aberrant β -catenin	*P* value	N-cadherin+	*P* value	E-cadherin-	*P* value
PRMT1						
**+**	37/37 (100)	1	58/69 (84.1)	0.878	20/66 (30.3)	0.468
**-**	36/36 (100)		40/47 (85.1)		11/46 (24.0)	
ZEB1						
**+**	21/21 (100)	1	33/37 (89.2)	0.380	12/36 (33.3)	0.280
**-**	52/52 (100)		63/76 (83.0)		17/73 (23.6)	
RUNX1						
**+**	46/46 (100)	1	65/70 (92.8)	0.003*	14/70 (20.0)	0.019*
**-**	26/26 (100)		32/45 (71.1)		17/42 (40.5)	
TWIST1						
**n+/−c**	20/20 (100)	1	38/46 (82.6)	0.563	14/46 (30.4)	0.528
**c**	52/52 (100)		58/67 (86.6)		16/64 (25.0)	
**-**	1/1 (100)		2/3 (66.5)		1/2 (50.0)	

Abbreviations: PRMT1 protein arginine methyltransferase 1; ZEB1, Zinc Finger E-Box Binding Homeobox 1; RUNX1, Runt-related transcription factor 1; TWIST1, Twist Family BHLH Transcription Factor 1; n, nuclear; c, cytoplasmic; +, positive; −, negative. *Statistically significant *p*-value

### Association of PRMT1, Zeb1, RUNX 1 and TWIST 1 expression with prognosis

**Fig. 4:**
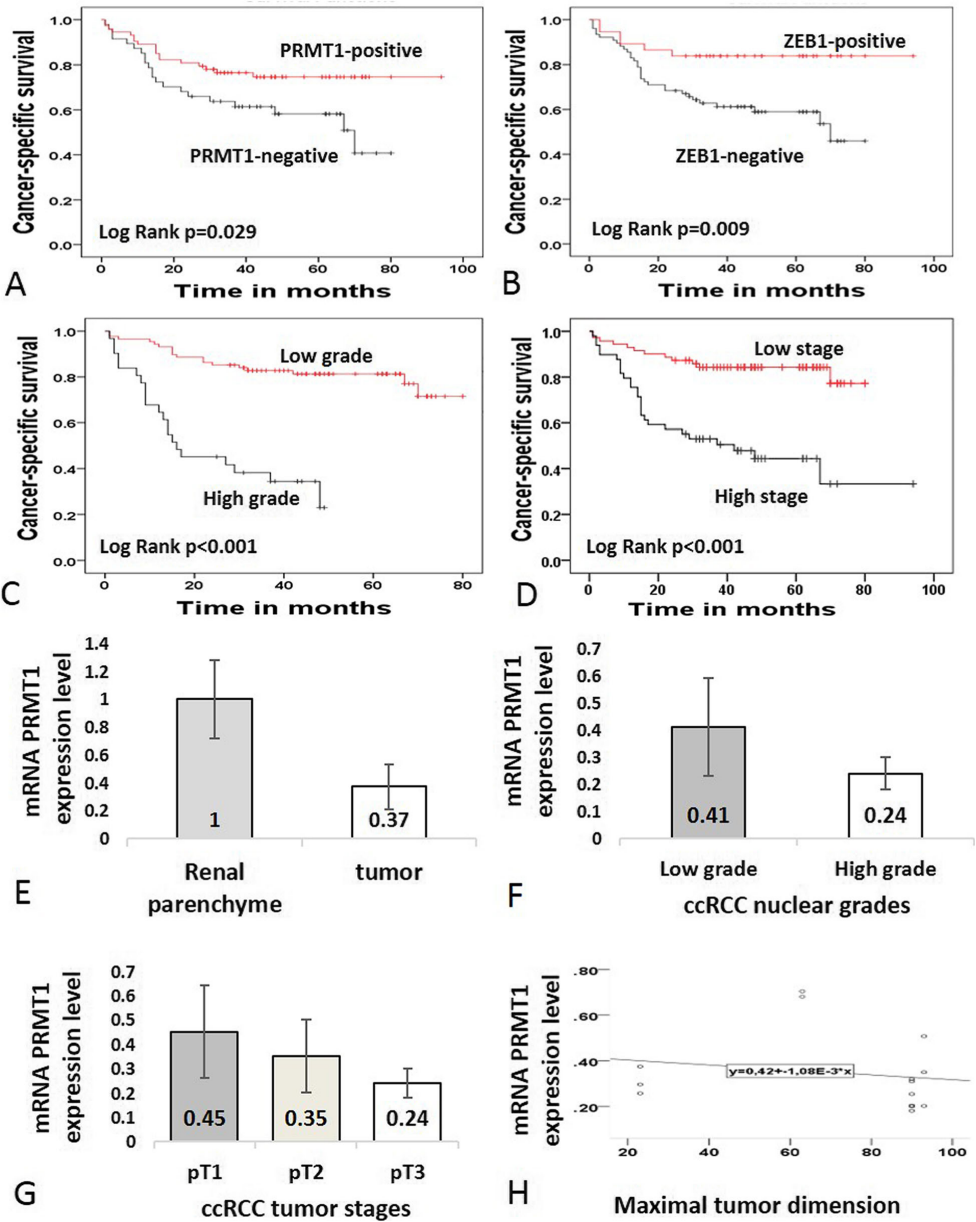
The survival analysis of ccRCC patients for the expressions of PRMT1 and ZEB1 in cancer cells, tumor grade and stage (**a**-**d**) and qRT-PCR analysis of PRMT1 in ccRCC (E-H). (**a**-**b)** Cancer-specific survival rates, revealed by Kaplan-Meier survival analysis, were higher in patients whose cancer cells were positive for both PRMT1 and ZEB1, than those who were negative for both of the markers, (**c**) Cancer-specific survival rate was lower in patients with high-grade carcinomas, when compared to the patients with low-grade carcinomas, (**d**) Cancer-specific survival rate was lower in patients with high stage tumors, in comparison to the patients with low stage tumor disease and partial nephrectomy; (**e**) mRNA PRMT1 expression was decreased in tumor tissue, related to adjacent non-tumor parenchyma, (**f**) Relative mRNA PRMT1 expression decrease was observed in high-grade tumors, when compared to low grade tumors; (**g**) Gradual decrease of mRNA PRMT1 was observed by each increase of tumor stage; (**h**) The graph illustrates the tendency of mRNA PRMT1 expression level decrease by the increase in tumor size; Relative mRNA expression levels are presented with mean values (+/− 1 SD)

During the follow-up time, disease-related death was documented in 48 (23.1%) patients. Diagnosed RCC types in patients who died of cancer related disease were as following: 39 (80%) ccRCC, 1(2%) pRCC I, 4(8.2%) pRCC II, 2 (4%) chRCC and 2 (4%) CDCs. One patient with oncocytoma died of an unrelated cause. Kaplan–Meier survival analyses revealed that PRMT1, ZEB1, nuclear grade, and tumor stage were significant prognostic factors in ccRCC. In particular, the expression of PRMT1 and ZEB1 in ccRCC, as well as low-nuclear tumor grade and low-tumor stage were significantly associated with better cancer-specific survival (*p* = 0.029, *p* = 0.009, *p* < 0.001 and *p* < 0.001, respectively) (Fig. 4 a-d; Table 8). Moreover, patients who died of ccRCC showed homogenous loss of PRMT1 in 44.7%, while 41.8% had homogenous loss of ZEB1 (*p* = 0.044, *p* = 0.009, respectively) (Table 9). RUNX1 and TWIST1 and patients’ gender didn’t show a correlation with cancer-specific survival (Table 8).

**Table 8:** Survival analysis. Mean survival of the patients within all types (*n* = 194) and ccRCC (*n* = 120); univariate and multivariate cancer-specific survival in the patients with ccRCC according to analyzed immunohistochemical markers and clinic-pathologic parameters

Prognostic factors	Cancer-specific survival (months)							
					Univariate		Multivariate	
	All types mean (95% CI)	*p*-value	ccRCC mean (95% CI)	*p*-value	Hazard ratio (95% CI)	*p*-value	Hazard ratio (95%CI)	*p*-value
PRMT1								
positive	78 (73–84)	0.009*	74 (66–82)	0.029*	0.504 (0.269–0.947) Ref.	0.033*	0.622 (0.326–1.188)	0.150
negative	56 (48–63)		51 (42–61)					
ZEB1								
positive	83 (76–91)	0.011*	80 (70–90)	0.009*	0.344 (0.140–0.801)	0.014*	0.367 (0.152–0.889)	0.026
negative	68 (61–74)		52 (45–60)					
RUNX1								
positive	75 (68–82)	0.937	67 (60–74)	0.506	0.872 (0.463–1.644)	0.673	N/A	
negative	69 (61–78)		57 (48–66)					
TWIST1								
positive	73 (68–78)	0.886	67 (60–74)	0.878	1.168 (0.159–8.553)	0.879	N/A	
negative	54 (34–75)		54 (34–75)					
Grade	N/A							
Low			67 (62–72)	<0.001*	5.802 (3.000–11.222)	<0.001*	6.284 (3.175–12.438)	<0.001*
High			25 (18–32)					
Stage	N/A							
Low			70 (63–76)	<0.001*	1.062 (0.849–1.330)	0.597	1.187 (0.912–1.547)	0.203
High			48 (36–60)					
PN			63 (51–74)					
Gender								
Male	75 (66–83)	0.686	58 (51–65)	0.736	1.123 (0.569–2.218)	0.737	N/A	
Female	72 (66–78)		70 (58–81)					

Abbreviations: *PRMT*1 protein arginine methyltransferase 1, *ZEB*1 Zinc Finger E-Box Binding Homeobox 1, *RUNX*1 Runt-related transcription factor 1, *TWIST*1 Twist Family BHLH Transcription Factor 1, *CI* confidence interval, *PN* partial nephrectomy, *ref*. referent value. *Statistically significant *p*-value

**Table 9:** The comparation of PRMT1 and ZEB1 expression heterogeneity and outcome of the patients with ccRCC

Prognostic marker	PRMT1	p value	ZEB1	*p* value
	Dead		Dead	
homogenous positive	4/24 (16.7)	0.044*	0/4 (0.0)	0.009*
heterogenous	14/49 (28.6)		5/32 (15.6)	
homogenous negative	21/47 (44.7)		33/79 (41.8)	

Abbreviations: PRMT1, protein arginine methyltransferase 1; ZEB1, Zinc Finger E-Box Binding Homeobox 1; *Statistically significant *p*-value

### qRT-PCR PRMT1 expression in cRCC, chRCC, and pRCC

A decrease of relative PRMT1 mRNA expression level was detected in tumor tissue compared to adjacent renal parenchyma (Fig. 4 e). Furthermore, relative PRMT1 mRNA expression level was lower in ccRCC than in chRCC and pRCC. However, ccRCC samples with nuclear grade II and lower tumor stages (I and II), had higher PRMT1 mRNA expression level (Fig. 4 f-g). In addition, we observed a tendency to decrease in PRMT1 mRNA expression level along with the increase of tumor size (Fig. 4 h). In these samples, PRMT1 IHC expression was observed in all three analyzed RCC types. In two cases of ccRCC with the highest grade, stage and with the largest tumor dimension relative mRNA PRMT1 expression was the lowest and PRMT1 IHC expression was homogenous negative and heterogenous. Interestingly, both cases of pRCC, type I expressed PRMT1 homogenously, although the relative PRMT1 mRNA level was different (Additional file 5: Table S4).

### mRNA PRMT1 expression in TCGA and GTEx cohort

**Fig. 5:**
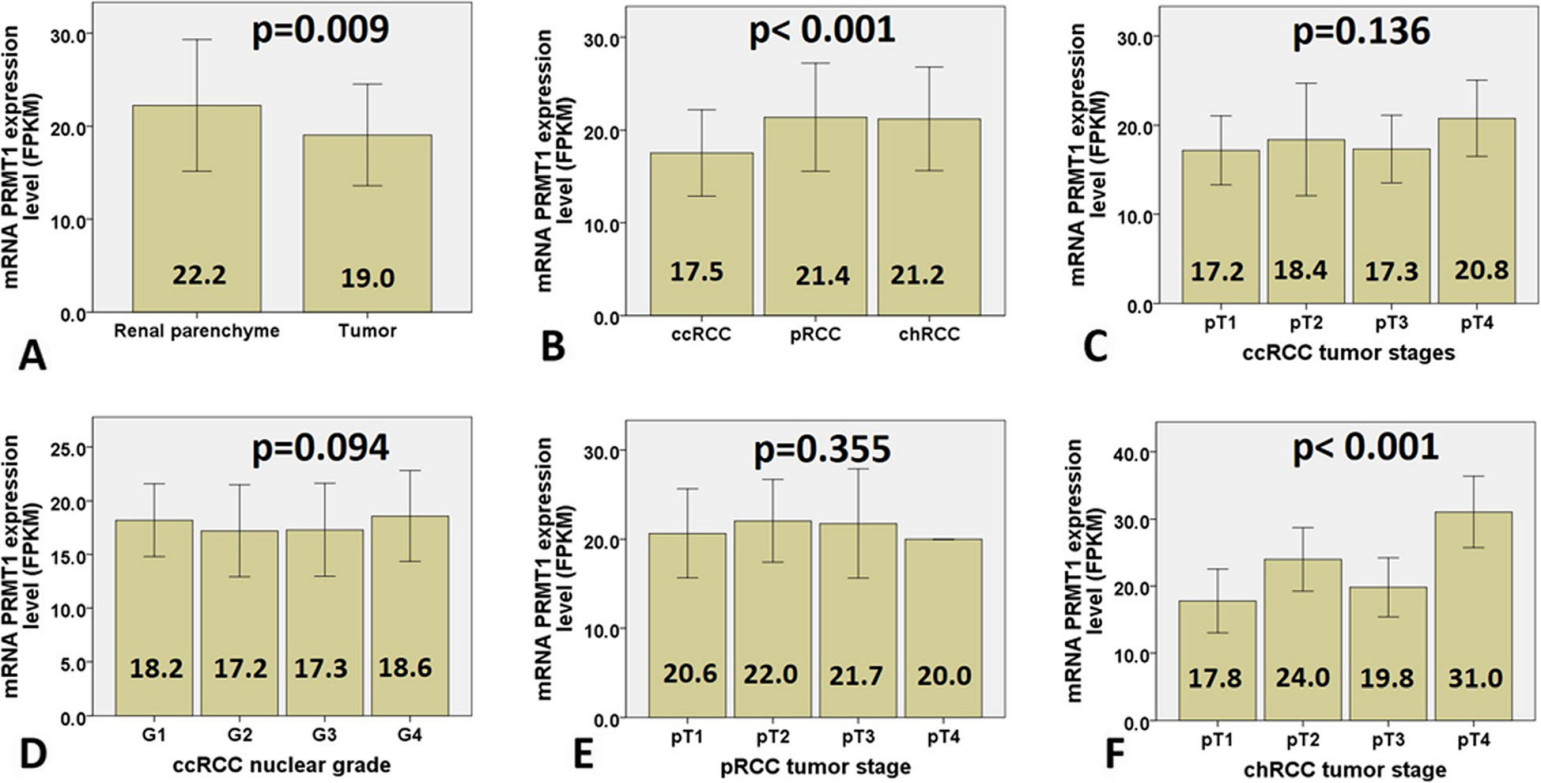
mRNA PRMT1 transcript profiling data (FPKM values) for TCGA cohorts relative to tumor tissue and tumor stage. **a** mRNA PRMT1 expression level was decreased in tumor tissue related to non-tumor parenchyma; (**b**) In tumor tissue, mRNA PRMT1 expression level was significantly lower in ccRCC, when compared to pRCC and chRCC; (**c**-**d**) However, mRNA PRMT1 expression level in ccRCC was not associated with tumor stage, neither with nuclear grade; (**e**) Likewise, mRNA PRMT1 expression level in pRCC was not associated with tumor stage; (**f**) On the other hand, mRNA PRMT1 expression increase was observed in stage IV of chRCC; mRNA expression levels are presented with Fragments Per Kilobase Million values (FPKM) (+/− 1 SD). Statistically significant difference *p* < 0.05

The results of mRNA PRMT1 expression analysis in the TCGA cohort are presented in Fig. 5. mRNA PRMT1 expression level in tumor tissue was significantly lower than that in renal parenchyma (*p* = 0.009; Fig. 5a). In tumor tissue, mRNA PRMT1 expression level was significantly lower in ccRCC, when compared to pRCC and chRCC (*p* < 0.001; Fig. 5b). However, mRNA PRMT1 expression level in ccRCC was not associated with tumor stage or grade, neither was associated with tumor stage of pRCC (*p* = 0.136; *p* = 0.094; *p* = 0.355, respectively (Fig. 5 c-e). On the other hand, mRNA PRMT1 expression level in chRCC was significantly higher in stage IV (*p* < 0.001; Fig. 5 f).

## Discussion

RCT is a heterogeneous group of neoplasms, with different biological behavior and prognosis [1]. Thus, persistent efforts to predict outcome in patients with renal neoplasms and to diagnose RCT precisely, have led scientists to investigate various biological markers. Furthermore, it has been widely accepted that epigenetic regulation plays an important role in many biological processes, including tumorigenesis [2].

PRMT1 is usually considered as an epigenetic molecular marker, expressed in many non-tumorous and tumorous tissues. PRMT1 immunopositivity in our study was equally observed in cortex and medulla of all cases used as a positive control. To the best of our knowledge, only one study reported IHC PRMT1 expression in renal tubules, without PRMT1 expression in glomerular structures, which could possibly be explained due to the use of different antibody PRMT1 clone [5].

The role of PRMT1 in tumorigenesis is still controversial and seems to depend on tumor type. Usually, it is overexpressed in tumors with high-grade morphology and high stage of the disease [14, 16]. Nevertheless, the protective effect of PRMT1 has been suggested in patients with gastric carcinomas, positively affecting survival and recurrence rates, especially in patients treated with adjuvant therapy [9]. Similarly, more frequent PRMT1 expression was observed in less advanced ccRCC, with low grade and low stage of the disease, while homogenous PRMT1 loss was detected in high grade and high stage ccRCC. Additionally, we found PRMT1 expression as more common in benign tumors, such as oncocytoma. Heterogeneous PRMT1, ZEB1, and TWIST1 expression are presumably related to ITH [24–26]. ITH is extensively studied in many tumor types, especially in ccRCC [25–29]. The significance of ITH is still arguable. Although it has been linked to aggressive tumor behavior, it seems that ITH appears early in carcinogenesis and therefore may not be necessarily associated with enhanced tumor malignancy [26, 28, 29]. Thus, heterogenous PRMT1 IHC expression in ccRCC may emphasize early acquired epigenetic diversity, which deserves further analyses.

Previously, other authors reported mRNA-PRMT1 level overexpression in cancerous tissue of the bladder, lung, breast, and glioma, when compared to non-tumor tissue. However, renal tumor and renal non-tumor tissues were not examined in their studies [5, 10, 13, 14]. Therefore, we analyzed mRNA PRMT1 expression level in TCGA/GTEx group in relation to our small sample. In both cohorts, mRNA PRMT1 expression level was downregulated in tumor tissue, when compared to counterparts, while the lowest level was observed in ccRCC type. Unlike in our study, mRNA PRMT1 expression level in ccRCC of TCGA cohort was not associated with tumor stage or tumor grade. Additionally, mRNA PRMT1 expression level up-regulation was associated only with high stage chRCC in the TCGA cohort. The observed differences in mRNA expression may be related to our small sample available for qRT-PCR analysis. Furthermore, different results obtained by IHC analysis in our cohort and mRNA expression analysis in TCGA group may be due to the variations in post-transcriptional mechanisms involved in mRNA PRMT1 translation into protein, as already suggested [30, 31]. In addition, gene expression variations in individual case within the same cancer subtype should be taken into account [32].

Our results imply PRMT1 as a ubiquitously expressed marker in the elements of the renal parenchyma. Therefore, PRMT1 may be one of “housekeeping” genes in renal tissue, required for the epigenetic regulation of normal kidney development [33]. Thus, according to results, we may speculate that any change in PRMT1 expression could lead to carcinogenesis and unfavorable prognosis of the patients.

EMT is considered responsible for renal tumorigenesis and progression, which is characterized by TWIST1 cytoplasmic localization, overexpression of N-cadherin, and loss of E-cadherin expression. In addition, ß-catenin dissociation from the membrane and its translocation to the cytoplasm or nucleus is the main feature of Wnt/ß-catenin EMT signaling pathway [17, 34–37]. EMT has been regulated by ZEB1, RUNX1 and TWIST1 transcription factors [11, 16, 38–40]. Furthermore, as an epigenetic regulator, PRMT1 can act as an EMT inducer or repressor in various cancers. Thus, in breast carcinoma, PRMT1 methylates ZEB1 promoter, which then induces EMT and therefore implies ZEB1 as a negative prognostic parameter [16]. On the other hand, PRMT1 activates RUNX1, that prevents EMT and therefore, RUNX1 serves as a tumor suppressor in various cancers [18, 39]. Finally, in non-small lung carcinoma, PRMT1 methylates TWIST1 and induces EMT related aggressive cancer behavior [11]. Following this, in more aggressive types of RCC and high-grade and/or high-stage tumors we expected frequent ZEB1 expression and co-expression with PRMT1. Unlike PRMT1, ZEB1 was not related to any of the analyzed clinicopathological parameters within different RCC types, but survival analysis revealed ZEB1 as an independent prognostic parameter of better survival of the patients with ccRCC. PRMT1 loss was often accompanied by ZEB1 loss in ccRCC. Thus, we assume that ZEB1 loss in ccRCC may be dependent on PRMT1 loss. This finding is different than those obtained in breast carcinoma and may be related to the different molecular mechanism of ZEB1 activity and specific PRMT1/ZEB1 interaction in RCT which haven’t been investigated so far. In this study, frequent homogenous PRMT1 and heterogenous ZEB1 IHC expression were observed in RO, considered to have benign behavior, while the majority of chRCC and RO was homogenously negative to ZEB1. This observation was confirmed on our small cohort of corresponding whole-mount sections. This may suggest that PRMT1/ZEB1 co-expression may be significant in RO tumorigenesis and may serve as a helpful IHC tool in the diagnosis of RO. However, this finding requires further investigation and confirmation on larger sample size, including conventional sections analysis. On the other hand, in less advanced carcinomas, we expected frequent RUNX1 expression and co-expression with PRMT1. Therefore, we may speculate that RUNX1/PRMT1 co-expression may be significant for the favorable behavior of renal neoplasms. Finally, in advanced carcinomas, we expected frequent TWIST1 cytoplasmic expression and co-expression with PRMT1. In ccRCC, loss of PRMT1 was associated with cytoplasmic TWIST1 expression, characteristic for aggressive ccRCC [36]. Therefore, we suggest that loss of PRMT1 in ccRCC could lead to TWIST1 cytoplasmic expression and more aggressive behavior. Moreover, two cases of high-grade ccRCC with sarcomatoid features showed homogenous PRMT1/ZEB1 loss and TWIST1 localization in the cytoplasm. This observation may be of great importance, that should be evaluated on larger cohort of RCT with sarcomatoid and/or rhabdoid change and compared to other high-grade RCC. EMT Wnt/ß-catenin pathway markers were not associated with PRMT1, ZEB1 or TWIST1 expression. Only RUNX1 was associated with N-cadherin overexpression. Our results suggest, that retention of PRMT1 expression may be a significant feature of less advanced ccRCC, while PRMT1 homogenous loss could be specific for high grade and high stage ccRCC. The role of PRMT1 in RCT, as an epigenetic marker, is barely related to WNT/ß-catenin-mediated EMT processes, since neither of EMT related cadherins or ß-catenin were associated with PRMT1 expression. Another possibility to explain the role of PRMT1 in various RCT emerge from the previous suggestions that PRMT1 indirectly, through different transcription factors, may affect tumor cell growth or cell apoptosis. Overall, this experimental study represents the first step of the future research that will evaluate the role of PRMT1, as a marker of favorable prognosis in ccRCC and its role in carcinogenesis of RCT.

## Conclusions

IHC expression of PRMT1 may be a valuable marker of low grade and low stage ccRCC, while homogenous PRMT1 loss may be significant for high grade and high stage ccRCC. Heterogenous PRMT1 IHC expression may emphasize intratumor heterogeneity which should be extensively examined in further studies. Loss of PRMT1 and/or ZEB1 IHC expression could be an indicator of poor prognosis in patients with ccRCC. As an epigenetic regulator, PRMT1 may be involved in other mechanisms of RCT development unrelated to EMT, such as regulation of the cell cycle or apoptosis, which remain to be clarified in the future.

### Limitations of this current study

Our study has several innate limitations to note. First, our small sample size of non-ccRCC tumor types with their retrospective nature was a major limitation. Other RCT types are relatively rare compared to ccRCC, especially MLCRN-LMP and CDC. Therefore, the main discussion of our paper relied on ccRCC, RO and chRCC. However, this is an experimental study, and the results of the current analysis on TMA should be confirmed in a larger multi-institutional study and using whole-mount sections. Furthermore, quantification of mRNA expression level could not be performed for all tumor types and for all markers, because of the lack of tissues and reagents for qRT-PCR analysis. Our hypothesis should, therefore, be further confirmed by obtaining tissue for other tumor types.

## Supplementary information

**Additional file 1: Figure S1.** Representative TMA microscopic photographs of PRMT1 and ZEB1 expression in ccRCC, chRCC and RO. (A-C) Strong heterogenous nuclear expression of PRMT1 and (D-F) homogenous negative ZEB1 staining in ccRCC, (G-L) Homogenous negative PRMT1 and ZEB1 in chRCC, (M-R) Strong homogenous positive nuclear PRMT1 expression and heterogenous nuclear ZEB1 expression in RO. Original magnification, × 40. Abbreviation: TMA, tissue microarray

**Additional file 2: Table S1.** mRNA PRMT1 expression level (FPKM) and clinical characteristics for all patients and their tumors

**Additional file 3: Table S2.** Patients characteristics in analyzed TCGA cohort

**Additional file 4: Table S3.** PRMT1 and ZEB1 IHC expression on TMA and corresponding whole-mount sections

**Additional file 5: Table S4.** Relative mRNA PRMT level and IHC PRMT1 expression in analyzed RCC

## Data Availability

The most data generated or analyzed during this study are included in this article and its supplementary information files.

## Abbreviations

AJCC: American Joint Committee on Cancer
CDC: collecting duct carcinomas – Bellini
cDNA: complementary DNA
chRCC: chromophobe renal renal cell carcinoma
DAP: diaminobenzidine
DNA: Deoxyribonucleic acid
EMT: epithelial to mesenchymal transition
FFPE: Formalin-Fixed Paraffin-Embedded
FPKM: Fragment Per Kilo Base Million
GTEx: Genotype-Tissue Expression
H&E: hematoxylin and eosin
IHC: immunohistochemical
ISUP: International Society of Urological Pathology
miRNA: microRNA
MLCRN-LMP: multilocular cystic renal cell neoplasms of low malignant potential
pRCC I: papillary RCC type I
pRCC II: papillary renal cell carcinoma type II
PRMT1: Protein arginine methyltransferase-1
qRT-PCR: Real-Time Quantitative Reverse Transcription Polymerase Chain Reaction
RCC: renal cell carcinomas
RCT: renal cell tumors
RNA: ribonucleic acid
RO: renal oncocytomas
RUNT1: Runt-related transcription factor 1
TCGA: The Cancer Genome Atlas
TMA: tissue microarray
TNM: tumor–node–metastasis
TWIST1: Twist Family BHLH Transcription Factor 1
ZEB1: Zinc Finger E-Box Binding Homeobox 1
