# Supplementary figures and images for: PRMT1 expression in renal cell tumors- application in differential diagnosis and prognostic relevance
Source: Diagn Pathol. 2019 Oct 26;14:120. doi: 10.1186/s13000-019-0901-6 (PMC6815371; doi:10.1186/s13000-019-0901-6)

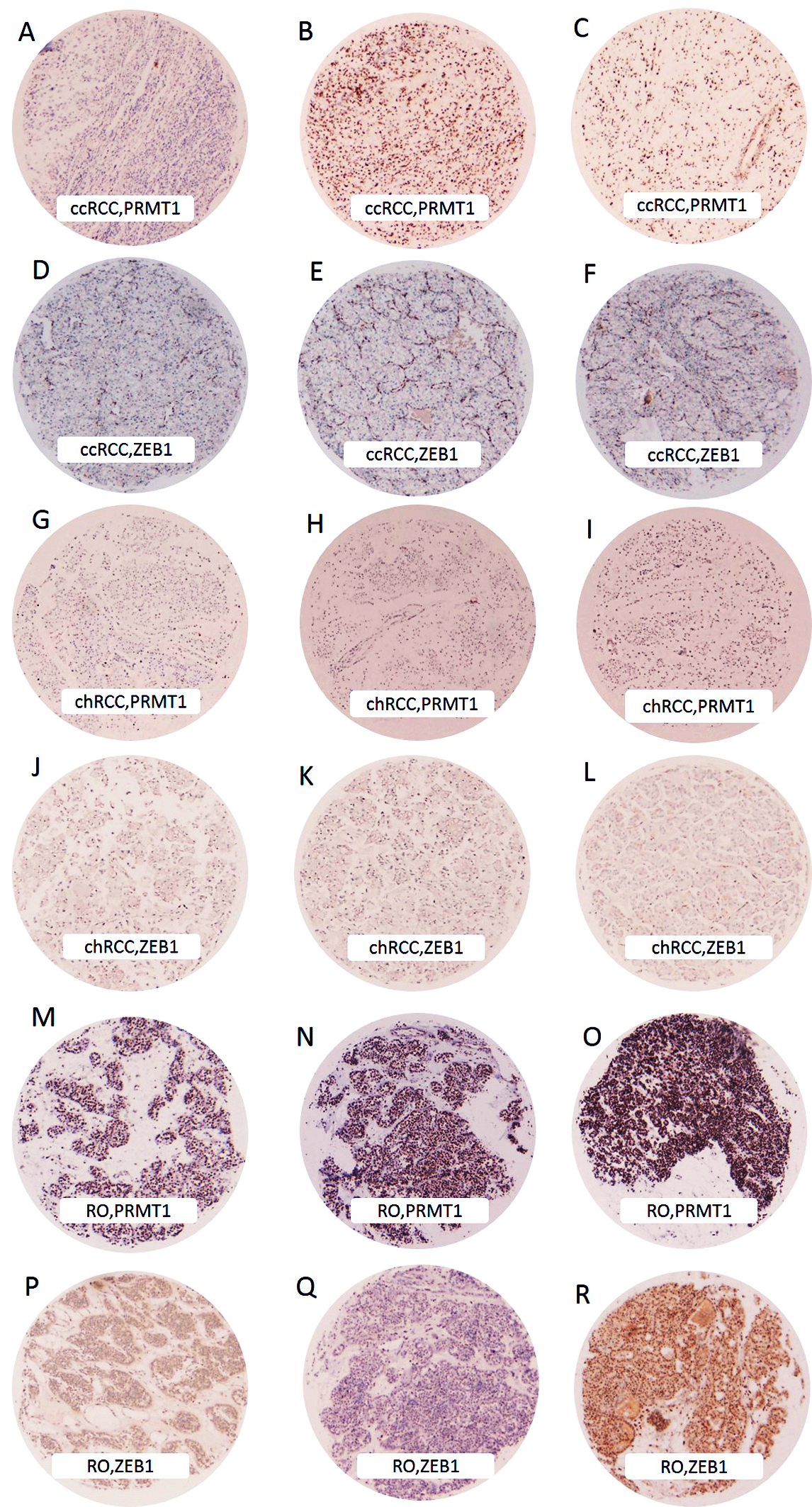

Supplement: Supplementary file 1 — Additional file 1: Figure S1. Representative TMA microscopic photographs of PRMT1 and ZEB1 expression in ccRCC, chRCC and RO. (A-C) Strong heterogenous nuclear expression of PRMT1 and (D-F) homogenous negative ZEB1 staining in ccRCC, (G-L) Homogenous negative PRMT1 and ZEB1 in chRCC, (M-R) Strong homogenous positive nuclear PRMT1 expression and heterogenous nuclear ZEB1 expression in RO. Original magnification, × 40. Abbreviation: TMA, tissue microarray [file 13000_2019_901_MOESM1_ESM.jpg]
