# Supplementary material for: PRMT1 expression in renal cell tumors- application in differential diagnosis and prognostic relevance
Source: Diagn Pathol. 2019 Oct 26;14:120. doi: 10.1186/s13000-019-0901-6 (PMC6815371; doi:10.1186/s13000-019-0901-6)
Supplement: Supplementary file 3 — Additional file 3: Table S2. Patients characteristics in analyzed TCGA cohort [file 13000_2019_901_MOESM3_ESM.docx]

**Additional file 3: Table S2**. Patients characteristics in analyzed TCGA cohort

|  | | **Histopathological subtypes of RCC TCGA cochort** | | | |
| --- | --- | --- | --- | --- | --- |
|  | ccRCC | | pRCC | chRCC | Total |
| **N** (%) | 528 (60.2) | | 285 (32.5) | 64 (7.2) | 877 (100) |
| **Age,** mean ±SD (years) | 60.1± 12.1 | | 61.7 ±11.9 | 51.6 ± 13.9 | 60.2±12.4 |
|  |  | |  |  |  |
| **Gender** N (%**)** |  | |  |  |  |
| Male | 344 (58.2) | | 209 (35.4) | 38 (6.4) | 591 (100) |
| Female | 184 (64.3) | | 76 (26.6) | 26 (9.1) | 286 (100) |
| **Grade** N (%) |  | |  |  |  |
| I | 11 (2.1) | |  |  | 11 (100) |
| II | 209 (39.6) | | N/A | N/A | 209 (100) |
| III | 198 (37.5) | |  |  | 198 (100) |
| IV | 71 (13.4) | |  |  | 71 (100) |
| N/A | 39 (7.4) | |  |  | 39 (100) |
| **Stage** N (%) |  | |  |  |  |
| I | 249 (55.7) | | 179 (40.0) | 19 (4.2) | 447 (100) |
| II | 64 (53.8) | | 30 (25.2) | 25 (21.0) | 119 (100) |
| III | 173 (70.3) | | 55 (22.3) | 18 (7.3) | 246 (100) |
| IV | 9 (34.6) | | 15 (57.7) | 2 (7.7) | 26 (100) |
| N/A | 33 (94.3) | | 2 (5.7) | 0 (0.0) | 35 (100) |

Abbreviations: TCGA, The Cancer Genome Atlas; RCC, Renal cell carcinomas; ccRCC,clear cell renal cell carcinomas; pRCC ,papillary renal cell carcinoma type; chRCC, chromophobe renal cell carcinoma; N, number; SD, standard deviation; N/A, not applicable; ISUP (International Society of Urological Pathology) nuclear grading system and updated American Joint Committee on Cancer (AJCC) tumor–node–metastasis (TNM) classification 8^th^ edition for tumor staging were used.
