# Supplementary material for: PRMT1 expression in renal cell tumors- application in differential diagnosis and prognostic relevance
Source: Diagn Pathol. 2019 Oct 26;14:120. doi: 10.1186/s13000-019-0901-6 (PMC6815371; doi:10.1186/s13000-019-0901-6)
Supplement: Supplementary file 4 — Additional file 4: Table S3. PRMT1 and ZEB1 IHC expression on TMA and corresponding whole-mount sections [file 13000_2019_901_MOESM4_ESM.docx]

**Additional file 4: Table S3**. PRMT1 and ZEB1 IHC expression on TMA and corresponding whole-mount sections.

| **Case No.** | **Tumor subtype** | **Nuclear grade** | **PRMT1**  **TMA/Whole-mount sections** | **ZEB1**  **TMA/ Whole-mount sections** |
| --- | --- | --- | --- | --- |
| 1. | ccRCC | low | Heterogenous (SC,M) | Heterogenous (M) |
| 2. | ccRCC | low | Heterogenous (SC,P) | Homogenous negative |
| 3. | ccRCC | low | Heterogenous (SC,M) | Heterogenous (SC,M) |
| 4. | ccRCC | low | Heterogenous (SC,P) | Homogenous negative |
| 5. | ccRCC | low | Homogenous positive | Heterogenous (SC) |
| 6. | ccRCC | low | Homogenous positive | Homogenous positive |
| 7. | ccRCC | low | Homogenous negative | Homogenous negative |
| 8. | ccRCC | high | Heterogenous (SC,P) | Homogenous negative |
| 9. | ccRCC | high | Homogenous negative | Homogenous negative |
| 10. | ccRCC | high (sarcomatoid features) | Homogenous negative | Homogenous negative |
| 11. | ccRCC | high | Heterogenous (M) | Heterogenous (SC) |
| 12. | ccRCC | high | Homogenous negative | Homogenous negative |
| 13. | ccRCC | high | Homogenous negative | Homogenous negative |
| 14. | ccRCC | high | Heterogenous (SC,M) | Heterogenous (SC,M) |
| 15. | ccRCC | high | Homogenous negative | Homogenous negative |
| 16. | pRCC, I | low | Homogenous positive | Homogenous negative |
| 17. | pRCC, I | low | Homogenous positive | Homogenous negative |
| 18. | pRCC, I | low | Heterogenous (SC,P) | Homogenous negative |
| 19. | pRCC, II | low | Homogenous positive | Homogenous negative |
| 20. | pRCC, II | low | Homogenous negative | Homogenous negative |
| 21. | pRCC, II | high | Homogenous negative | Homogenous negative |
| 22. | pRCC, II | high | Heterogenous (SC,M) | Homogenous negative |
| 23. | pRCC, II | high | Homogenous negative | Homogenous negative |
| 24. | pRCC, II | high | Heterogenous (M,P) | Homogenous negative |
| 25. | pRCC, II | high | Homogenous negative | Homogenous negative |
| 26. | chRCC | N/A | Homogenous negative | Homogenous negative |
| 27. | chRCC | N/A | Homogenous positive | Homogenous negative |
| 28. | chRCC | N/A | Heterogenous (P) | Homogenous negative |
| 29. | chRCC | N/A | Homogenous negative | Homogenous negative |
| 30. | chRCC | N/A | Homogenous negative | Homogenous negative |
| 31. | chRCC | N/A | Homogenous negative | Homogenous negative |
| 32. | chRCC | N/A | Homogenous negative | Homogenous negative |
| 33. | chRCC | N/A | Heterogenous (P,M) | Homogenous negative |
| 34. | chRCC | N/A | Heterogenous (SC) | Homogenous negative |
| 35. | chRCC | N/A | Homogenous negative | Homogenous negative |
| 36. | RO | N/A | Homogenous positive | Heterogenouos (SC) |
| 37. | RO | N/A | Homogenous positive | Homogenous positive |
| 38. | RO | N/A | Homogenous positive | Heterogenouos (SC,M) |
| 39. | RO | N/A | Homogenous positive | Heterogenouos (SC,M) |
| 40. | RO | N/A | Homogenous positive | Heterogenouos (SC,P) |
| 41. | RO | N/A | Homogenous positive | Heterogenouos (SC,M) |
| 42. | RO | N/A | Heterogenous (P) | Heterogenouos (SC,M) |
| 43. | RO | N/A | Homogenous positive | Homogenous positive |
| 44. | RO | N/A | Heterogenous (SC,M) | Homogenous negative |
| 45. | RO | N/A | Homogenous positive | Heterogenouos (SC) |

Abbreviations: No, number; ccRCC, clear cell renal cell carcinoma;PRCC, papillary renal cell carcinoma; chRCC, chromophobe renal cell carcinoma; RO, renal oncocytoma; TMA, tissue microarray; PRMT1, protein arginine methyltransferase 1; ZEB1, Zinc Finger E-Box Binding Homeobox 1; SC, subcapsular; M, middle; P, periphery; N/A, not applicable.
