# Supplementary material for: PRMT1 expression in renal cell tumors- application in differential diagnosis and prognostic relevance
Source: Diagn Pathol. 2019 Oct 26;14:120. doi: 10.1186/s13000-019-0901-6 (PMC6815371; doi:10.1186/s13000-019-0901-6)
Supplement: Supplementary file 5 — Additional file 5: Table S4. Relative mRNA PRMT level and IHC PRMT1 expression in analyzed RCC [file 13000_2019_901_MOESM5_ESM.docx]

**Additional file 5: Table S4**. Relative mRNA PRMT level and IHC PRMT1 expression in analyzed RCC.

| **ID** | **Tumor type** | **Stage** | **Grade** | **Gender** | **Maximal tumor dimension** | **mRNA PRMT1 expression level*** | **PRMT1 IHC staining** |
| --- | --- | --- | --- | --- | --- | --- | --- |
| **1.** | ccRCC | 1 | 2 | M | 23 | 0.31 | Homogenous positive |
| **2.** | ccRCC | 1 | 2 | F | 63 | 0.58 | Heterogenuos positive |
| **3.** | ccRCC | 2 | 2 | M | 93 | 0.35 | Homogenous positive |
| **4.** | ccRCC | 3 | 3 | F | 90 | 0.20 | Heterogenous positive |
| **5.** | ccRCC | 3 | 3 | M | 90 | 0.29 | Homogenous negative |
| **6.** | pRCC, type I | 3 | 2 | F | 70 | 0.31 | Homogenous positive |
| **7.** | pRCC, type I | 3 | 2 | F | 70 | 0.56 | Homogenous positive |
| **8.** | chRCC | 3 | 4 | F | 112 | 0.39 | Heterogenous positive |

Abbreviations: PRMT1, protein arginine methyltransferase 1; ccRCC, clear cell renal cell carcinomas; pRCC, papillary renal cell carcinoma; chRCC, chromophobe renal cell carcinoma; *Mean values of mRNA PRMT1 are presented; samples were run in triplicates and the mRNA expression levels were quantitatively analyzed and normalized to the level of glyceraldehyde 3-phosphate dehydrogenase (GAPDH) housekeeping gene.
